# Loss of *Cln5* causes altered neurogenesis in a mouse model of a childhood neurodegenerative disorder

**DOI:** 10.1242/dmm.029165

**Published:** 2017-09-01

**Authors:** Ekaterina Savchenko, Yajuvinder Singh, Henna Konttinen, Katarina Lejavova, Laura Mediavilla Santos, Alexandra Grubman, Virve Kärkkäinen, Velta Keksa-Goldsteine, Nikolay Naumenko, Pasi Tavi, Anthony R. White, Tarja Malm, Jari Koistinaho, Katja M. Kanninen

**Affiliations:** 1 A.I. Virtanen Institute for Molecular Sciences, University of Eastern Finland, 70211 Kuopio, Finland; 2 Department of Pathology, University of Melbourne, Parkville 3010, Australia; 3 Anatomy and Developmental Biology, Monash University, Clayton 3168, Australia; 4 Cell and Molecular Biology, QIMR Berghofer Medical Research Institute, Herston 4006, Australia

**Keywords:** Neurogenesis, Neuronal ceroid lipofuscinoses, Batten disease, Lysosomal storage disease, Stem cells, CLN5

## Abstract

Neural stem/progenitor cells (NPCs) generate new neurons in the brain throughout an individual's lifetime in an intricate process called neurogenesis. Neurogenic alterations are a common feature of several adult-onset neurodegenerative diseases. The neuronal ceroid lipofuscinoses (NCLs) are the most common group of inherited neurodegenerative diseases that mainly affect children. Pathological features of the NCLs include accumulation of lysosomal storage material, neuroinflammation and neuronal degeneration, yet the exact cause of this group of diseases remains poorly understood. The function of the CLN5 protein, causative of the CLN5 disease form of NCL, is unknown. In the present study, we sought to examine neurogenesis in the neurodegenerative disorder caused by loss of *Cln5*. Our findings demonstrate a newly identified crucial role for CLN5 in neurogenesis. We report for the first time that neurogenesis is increased in *Cln5*-deficient mice, which model the childhood neurodegenerative disorder caused by loss of *Cln5*. Our results demonstrate that, in *Cln5* deficiency, proliferation of NPCs is increased, NPC migration is reduced and NPC differentiation towards the neuronal lineage is increased concomitantly with functional alterations in the NPCs. Moreover, the observed impairment in neurogenesis is correlated with increased expression of the pro-inflammatory cytokine IL-1β. A full understanding of the pathological mechanisms that lead to disease and the function of the NCL proteins are critical for designing effective therapeutic approaches for this devastating neurodegenerative disorder.

## Abstract (precis)

**Editors’ choice:** This study demonstrates neurogenic alterations in the most common neurodegenerative disease of children and reports a novel function of the CLN5 protein.

## INTRODUCTION

Neurogenesis occurs throughout life in the mammalian brain (Altman, 1962), and is correlated with learning and memory functions. During this complex process, new neurons are generated in specific neurogenic niches in the brain, including the subgranular zone of the hippocampal dentate gyrus and the subventricular zone of the lateral ventricles. The newly generated neurons are derived from neuronal stem/progenitor cells (NPCs), which proliferate and produce neuronal and glial cells under physiological conditions. This dynamic process requires coordinated cell proliferation, apoptosis, migration, differentiation and functional integration. The regulation of neuronal development in the postnatal period is intricate and involves numerous intrinsic and extracellular factors, including growth factors and cytokines (Faigle and Song, 2013).

Neurogenic impairments occur in the aging brain and in several neurodegenerative diseases. During aging, the proliferation of NPCs is reduced, resulting in a reduction in the production of new neurons. However, the survival and differentiation of the limited number of newly born cells is thought to remain unaltered during the aging process (Shruster et al., 2010). There is increasing evidence demonstrating that alterations in neurogenesis occur in neurodegenerative disorders, including Alzheimer's disease, Parkinson's disease and many others (Winner and Winkler, 2015). In addition to a gradual loss of existing neuronal populations, impairments in the endogenous capacity for cell renewal are a common pathological feature of these diseases. In most instances, the exact mechanisms underlying neurogenic defects remain unknown. Moreover, contradictory reports exist as to whether neurogenesis is increased or reduced during neurodegenerative diseases.

Neuronal ceroid lipofuscinoses (NCLs) form the most common group of neurodegenerative disorders that primarily affect children. This group of inherited disorders is characterized by progressive blindness, difficulties in learning, motor impairment and premature death. NCL is caused by loss of the ceroid-lipofuscinosis neuronal (CLN) proteins, 13 of which have been identified to date (Mole and Cotman, 2015). Depending on the affected CLN protein, the age of disease onset and rate of progression varies, yet the ultimate outcome is the same. Pathological features of the NCLs include accumulation of lysosomal storage material, neuroinflammation and neuronal degeneration. The CLN5 disease form of NCL (Santavuori et al., 1973) has a mean age of onset of 5.6 years (Xin et al., 2010) and is caused by mutations in the *CLN5* gene. The CLN5 protein is a glycoprotein that does not share any apparent homology with other proteins. It is associated with lipid metabolism, myelination, protein transport and endosomal sorting (Cárcel-Trullols et al., 2015). Recently, it was shown that CLN5 might be important during embryonic development (Fabritius et al., 2014). However, the exact function of CLN5 remains unknown.

The present study sought to examine neurogenesis in a neurodegenerative disorder caused by loss of *Cln5*. We demonstrate for the first time the importance of CLN5 for neurogenesis. Neurogenic alterations are manifested by reduced proliferation, altered differentiation and impaired function of NPCs. Furthermore, increased neurogenesis was found to be associated with high levels of interleukin-1β (IL-1β) in the diseased brains, and altered IL-1β signaling was observed in NPCs *in vitro*. Our results suggest a newly identified role for CLN5 in neurogenesis.

## RESULTS

### *Cln5* deficiency increases hippocampal neurogenesis

**Fig. 1.**
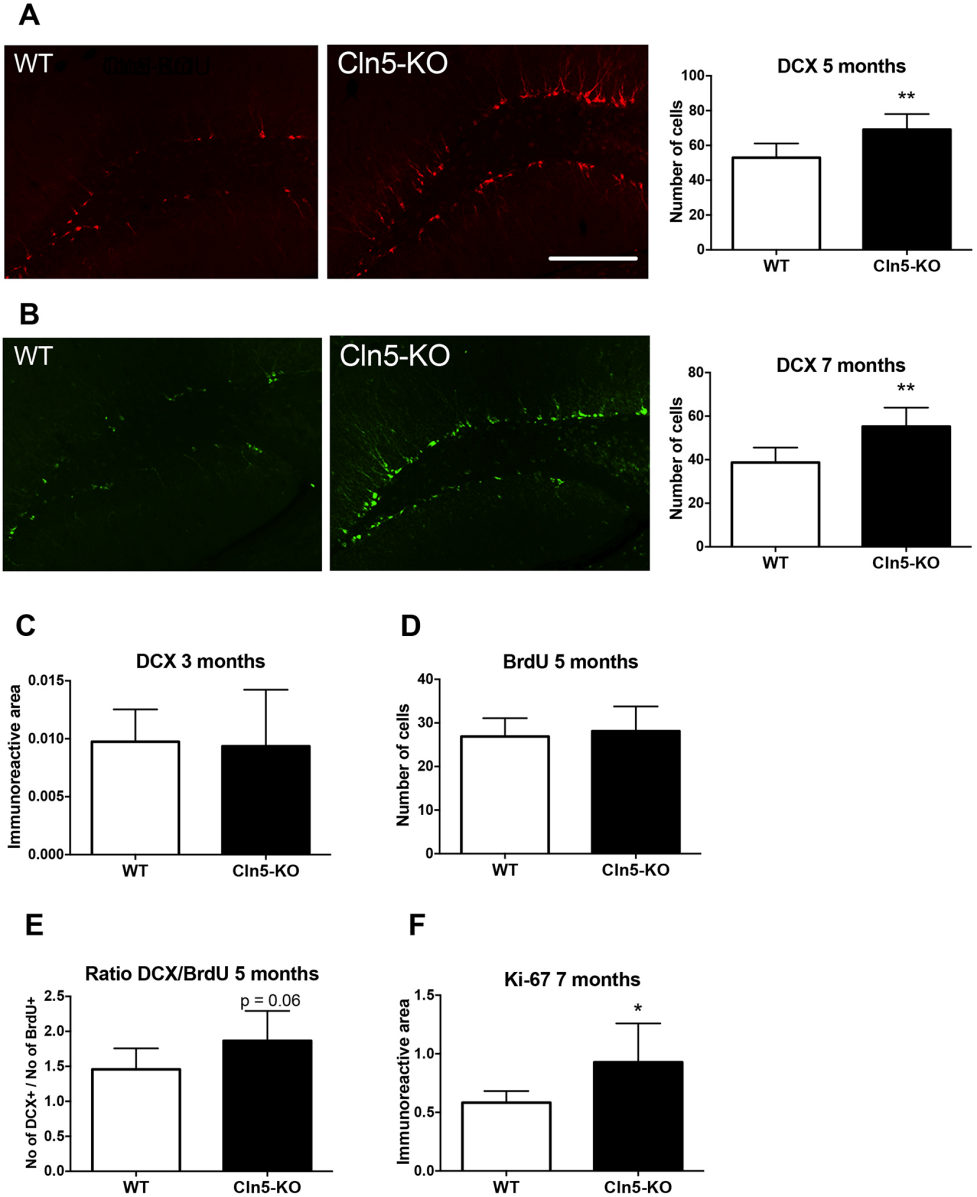
**Neurogenesis is increased in *Cln5*-deficient brains.** (A) Representative DCX immunostaining images from hippocampal dentate gyrus of 5-month-old WT and *Cln5*-KO mice. The graph shows the numbers of DCX-positive cells counted from 4-6 brain sections at 400-μm intervals from 6 animals of each genotype. **P<0.001. Scale bar: 200 μm. (B) Representative DCX immunostaining images from hippocampal dentate gyrus of 7-month-old WT and *Cln5*-KO mice. The graph shows the numbers of DCX-positive cells counted from 4-6 brain sections at 400-μm intervals from 6 animals of each genotype. **P<0.001. (C) The immunoreactive area occupied by DCX staining was quantified from 4-6 brain sections at 400-μm intervals from 7-10 mice of each genotype at the age of 3 months. (D) The number of BrdU-positive cells was calculated from 4-6 brain sections at 400 μm intervals of the hippocampal dentate gyrus of 5-month-old WT and *Cln5*-KO mice after 7 days of intraperitoneal BrdU injection. *N*=7/genotype. (E) The number of DCX-positive cells in the hippocampal dentate gyrus was calculated following 7 days of BrdU injection and is shown proportional to the number of BrdU-positive cells. *N*=7/genotype. (F) The immunoreactive area occupied by Ki-67 staining was quantified from 7-month-old WT and *Cln5*-KO lateral wall of the lateral ventricle. *N*=6-7/genotype. *P<0.05. The data in this figure are expressed as mean number of cells or the mean area occupied by the immunoreactivity in the dentate gyrus of the hippocampus±s.d. Statistical method: 2-tailed *t*-test.

To assess whether the disease caused by loss of *Cln5* is associated with neurogenic alterations *in vivo*, brains from *Cln5*-knockout (KO) and wild-type (WT) mice at various ages were processed for immunohistochemistry. Quantification of the expression of doublecortin (DCX), a marker expressed by neuronal precursor cells and immature neurons, revealed a 23% increase in the number of newly born neurons in the hippocampal dentate gyrus at the age of 5 months (Fig. 1A) and a 29% increase at the age of 7 months (Fig. 1B). However, changes in the number of DCX-positive cells were not observed at the age of 3 months (Fig. 1C). Quantification of hippocampal dentate gyrus bromodeoxyuridine (BrdU) labeling following a 7-day BrdU administration revealed that the numbers of proliferating cells were similar in WT and *Cln5*-KO dentate gyrus at the age of 5 months (Fig. 1D). The ratio of DCX-positive cells to BrdU-labeled cells trended towards an increase in the *Cln5*-KO dentate gyrus (Fig. 1E). In the subventricular zone of the lateral ventricles of *Cln5*-deficient mice, the number of Ki-67 (marker of proliferation)-positive cells was increased by 38% at the age of 7 months (Fig. 1F). This indicates that cell proliferation is increased in the subventricular zone in *Cln5*-KO mice. Taken together, these results indicate that *Cln5* deficiency alters neurogenesis in a time-dependent fashion, and that the number of newly forming precursors and immature neurons is significantly increased in *Cln5*-KO mice.

### Proliferation is increased in *Cln5* deficiency

**Fig. 2.**
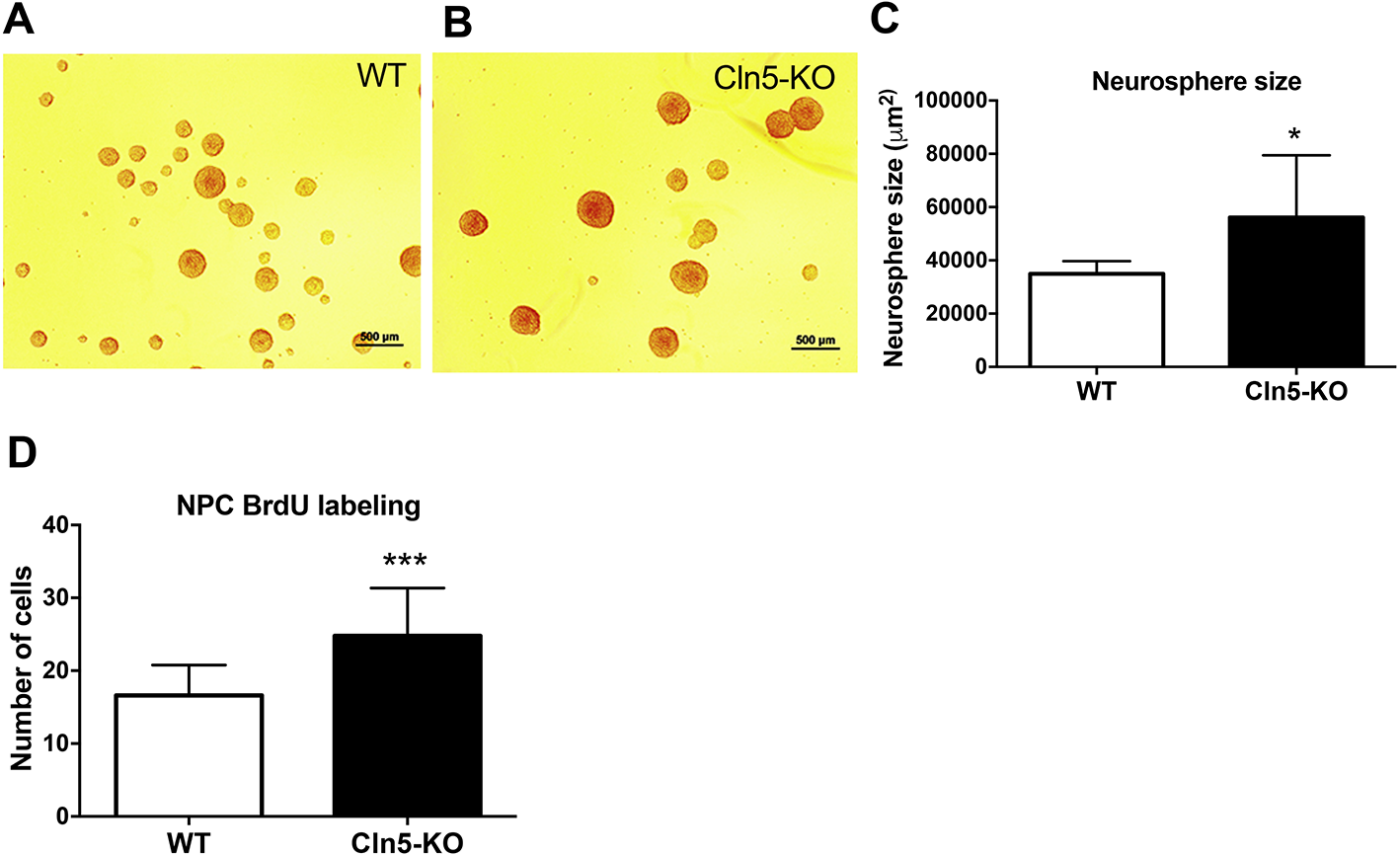
**Proliferation is increased in *Cln5* deficiency.** (A,B) Representative images of WT and *Cln5*-KO neurospheres that were formed after single NPCs were allowed to proliferate in culture for 5 days. (C) The volumes of the formed neurospheres were determined from 6-7 replicate images. Data are expressed as neurosphere size in μm^2^±s.d. *P<0.05. (D) Proliferation of NPCs was assessed by incubating cells with BrdU for 4 h, after which cell clusters were dissociated, left to grow overnight and immunostained with BrdU antibody and nuclear bisBenzimide stain. The numbers of cells positive for BrdU staining were calculated from 5 images taken from 5 replicate wells each. The data are expressed as mean number of cells±s.d. ***P<0.001. Statistical method: 2-tailed *t*-test.

Increased neurogenesis can occur through several mechanisms, including accelerated proliferation. To understand whether the observed increase in neurogenesis in *Cln5*-KO brains was related to the increased proliferation of cells, we next applied an *in vitro* approach to study the phenotype of NPCs isolated from *Cln5*-KO hippocampi. NPCs cultured from embryonic hippocampi grow as neurospheres and serve as an *in vitro* model for the study of neurogenesis. To examine the effect of *Cln5* deficiency on the proliferation of NPCs isolated from the hippocampi, we cultured single NPCs in the presence of growth factors and assessed sphere size and BrdU incorporation by pulse labeling. *Cln5*-deficient NPCs formed larger neurospheres in comparison to WT neurospheres (Fig. 2A,B). Quantification of the sphere size revealed that *Cln5*-KO spheres were approximately 38% larger than the WT controls after 5 days in culture (Fig. 2C). BrdU pulse labeling experiments also demonstrated that the number of proliferated *Cln5*-deficient cells was approximately 32% higher compared to WT cells (Fig. 2D). Taken together, these results demonstrate that *Cln5* deficiency causes increased proliferation in the neurogenic niche *in vivo* and of NPCs *in vitro*.

### Loss of *Cln5* does not increase apoptosis in the hippocampi

**Fig. 3.**
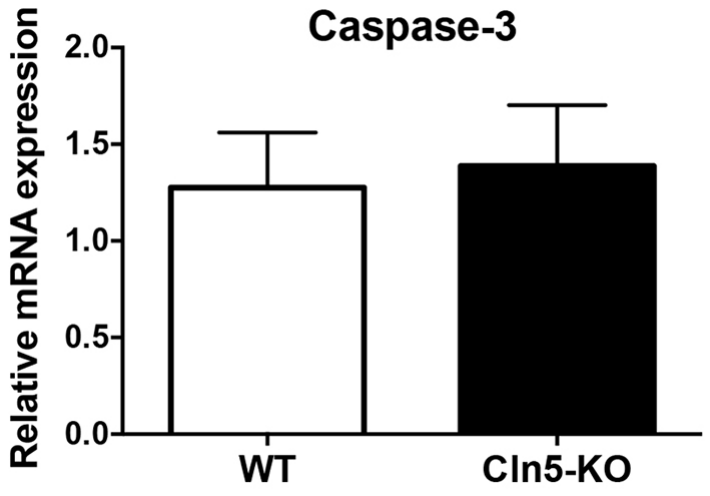
**Loss of *Cln5* does not affect the degree of apoptosis in the hippocampi.** Relative expression of mRNA encoding caspase-3 in WT (*n*=5) and *Cln5*-KO (*n*=7) hippocampi were analyzed by qRT-PCR. The mRNA expression level is presented as mean relative expression level normalized to GAPDH±s.d. Statistical method: 2-tailed *t*-test.

To ensure that the observed increase in neurogenesis in *Cln5*-deficient mice was not related to alterations in the degree of apoptosis, hippocampal caspase-3 levels were assessed by immunohistochemical stainings and quantitative real-time polymerase chain reaction (qRT-PCR). The mRNA expression level of caspase-3 was not altered in the hippocampi of 7-month-old *Cln5*-KO mice (Fig. 3). Similarly, the degree of cleaved caspase-3 immunoreactivity or TUNEL staining was not altered in the hippocampi when WT mouse brain sections were compared to those of *Cln5*-deficient brains (data not shown). These results suggest that the observed alterations in neurogenesis are not related to changes in hippocampal apoptosis caused by *Cln5* deficiency.

### *Cln5* deficiency alters the phenotype of neuronal progenitors

To further characterize the effects of *Cln5* deficiency on the phenotype of NPCs, we next examined the expression of *Cln5* in NPCs, the migration and differentiation of NPCs, and NPC neurite length.

**Fig. 4.**
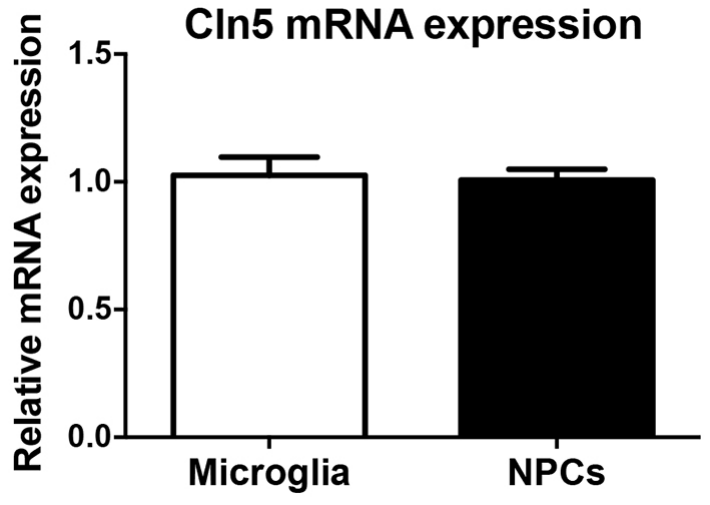
**Relative *Cln5* expression level in hippocampal NPCs.** Relative expression of mRNA encoding CLN5 in hippocampal neurospheres and primary microglia were analyzed by qRT-PCR. The mRNA expression level is presented as mean relative expression level normalized to GAPDH±s.d. Statistical method: 2-tailed *t*-test.

It has been reported that *Cln5* mRNA expression is approximately 4-fold higher in microglia than in neurons (Schmiedt et al., 2012). However, the expression of *Cln5* in hippocampal NPCs has not been measured. To quantify the expression level of *Cln5* mRNA in NPCs, we compared NPC expression to that of microglia using qRT-PCR. These analyses showed that *Cln5* is expressed at a similar level in NPCs compared to microglia (Fig. 4). The expression level of *Cln5* in NPCs suggests an important role of *Cln5* in neurogenesis.

**Fig. 5.**
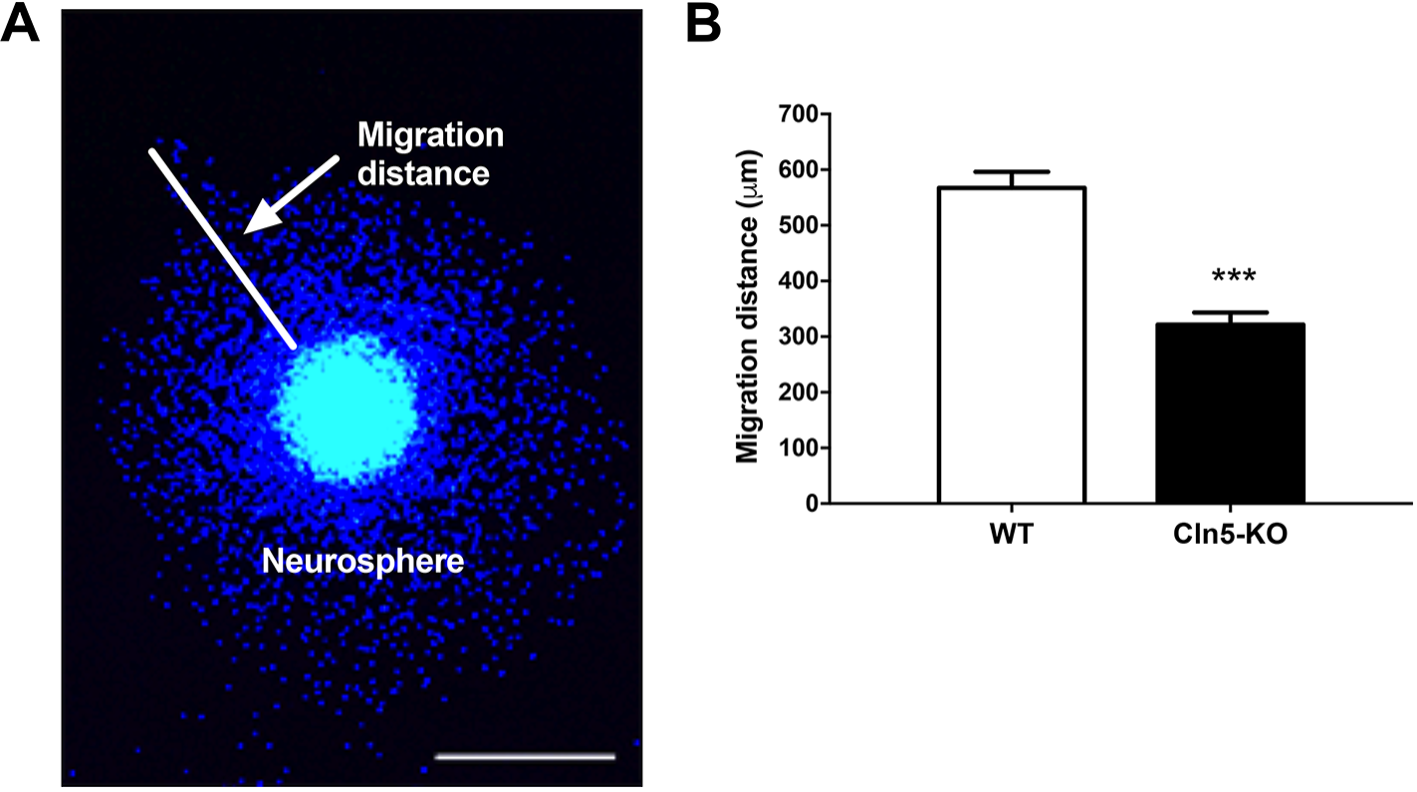
**Migration of *Cln5*-deficient NPCs is reduced.** (A) Representative image of migrating NPCs (DAPI staining). Scale bar: 500 μm. (B) Average migration distances of WT and *Cln5*-KO NPCs were measured from 10 replicate wells of each genotype and are shown as mean migration distance in μm±s.d. ***P<0.001. Statistical method: 2-tailed *t*-test.

To determine the effect of *Cln5* loss on the phenotype of hippocampus-derived NPCs, we first assessed NPC migration. In the absence of growth factors, single NPCs began to migrate away from the edges of the neurosphere and differentiate. The migration of NPCs was determined by measuring the distance traveled from the edge of the neurosphere by individual cells (Fig. 5A). In comparison to WT cells, the migration of *Cln5*-deficient NPCs was reduced by close to 40% at 7 days after plating (Fig. 5B). This reduction in the migration of *Cln5*-KO cells was also observed at 3 days after plating (data not shown).

**Fig. 6.**
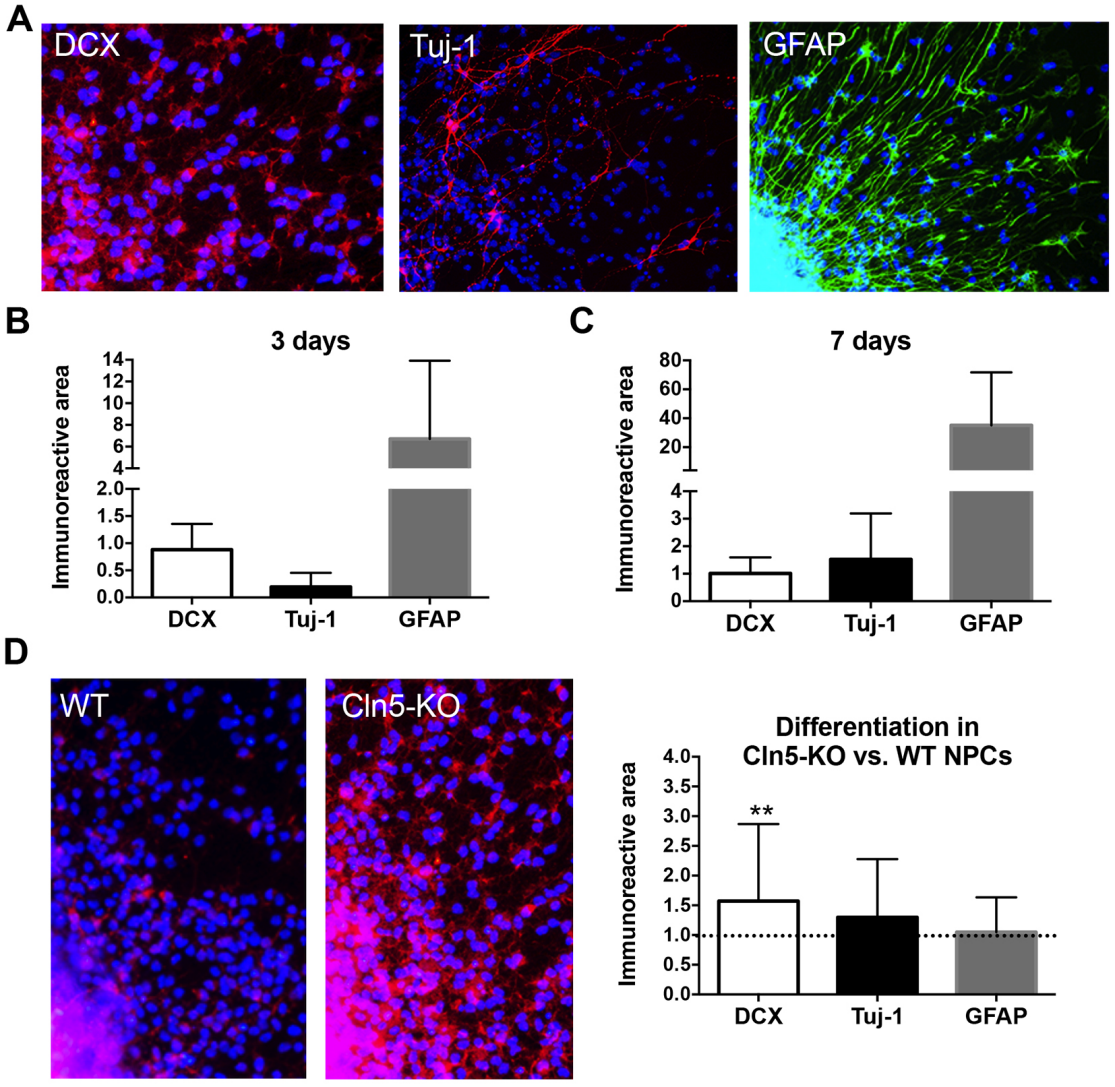
**Differentiation of *Cln5*-KO NPCs is altered.** (A) Representative images of the phenotype of differentiating WT NPCs after 7 days in culture. Staining with DCX, Tuj-1 and GFAP was used to visualize the phenotype of the cells. (B,C) The immunoreactive area occupied by DCX, Tuj-1 and GFAP staining was quantified from 8 replicate wells each from the periphery of the neurosphere area where the NPCs grow separately. The data are expressed as the mean relative area occupied by the immunoreactivity compared to DAPI immunoreactivity area±s.d. Results are normalized so that DCX receives a value of 1 to which GFAP and Tuj-1 are compared. (D) Representative images of WT and *Cln5*-KO NPC cultures immunostained with DCX after 3 days of differentiation. The immunoreactivity for each phenotypic marker was quantified from 8 replicate wells and is expressed as the mean area occupied by the immunoreactivity±s.d. Results are normalized so that the WT receives a value of 1 (not shown) and the columns represent *Cln5*-KO results relative to WT values. Statistical method: 2-tailed *t*-test.

In order to further understand the role of *Cln5* in neurogenesis, we next assessed the effect of *Cln5* loss on the differentiation of NPCs. NPC differentiation was assessed by immunocytochemical staining for DCX, neuron-specific class beta III tubulin (Tuj-1; a marker of mature neurons) and glial fibrillary acid protein (GFAP; a marker of astrocytes). Fig. 6 demonstrates the proportion of cells that are positive for each of the markers at 3 and 7 days after plating. After 3 days of differentiation, the proportion of immunoreactivity occupied by Tuj-1 staining is approximately 70% lower than that of DCX (Fig. 6B). Four days later, Tuj-1 staining is higher than that of DCX, indicating the maturation of the neuronal cells (Fig. 6C). The majority of the cells are positive for GFAP at both 3 and 7 days after differentiation. Comparison of the differentiation of WT NPCs to that of *Cln5*-deficient NPCs revealed that the proportion of both DCX- and Tuj-1-expressing cells was higher in the *Cln5*-KO cultures after 3 days of differentiation (Fig. 6D). The expression of DCX was increased 1.6-fold in the *Cln5*-deficient NPC cultures. The expression of Tuj-1 appeared to also be increased in the *Cln5*-deficient cells, but this difference was not statistically significant. Loss of *Cln5* did not alter the proportion of GFAP-positive cells.

**Fig. 7.**
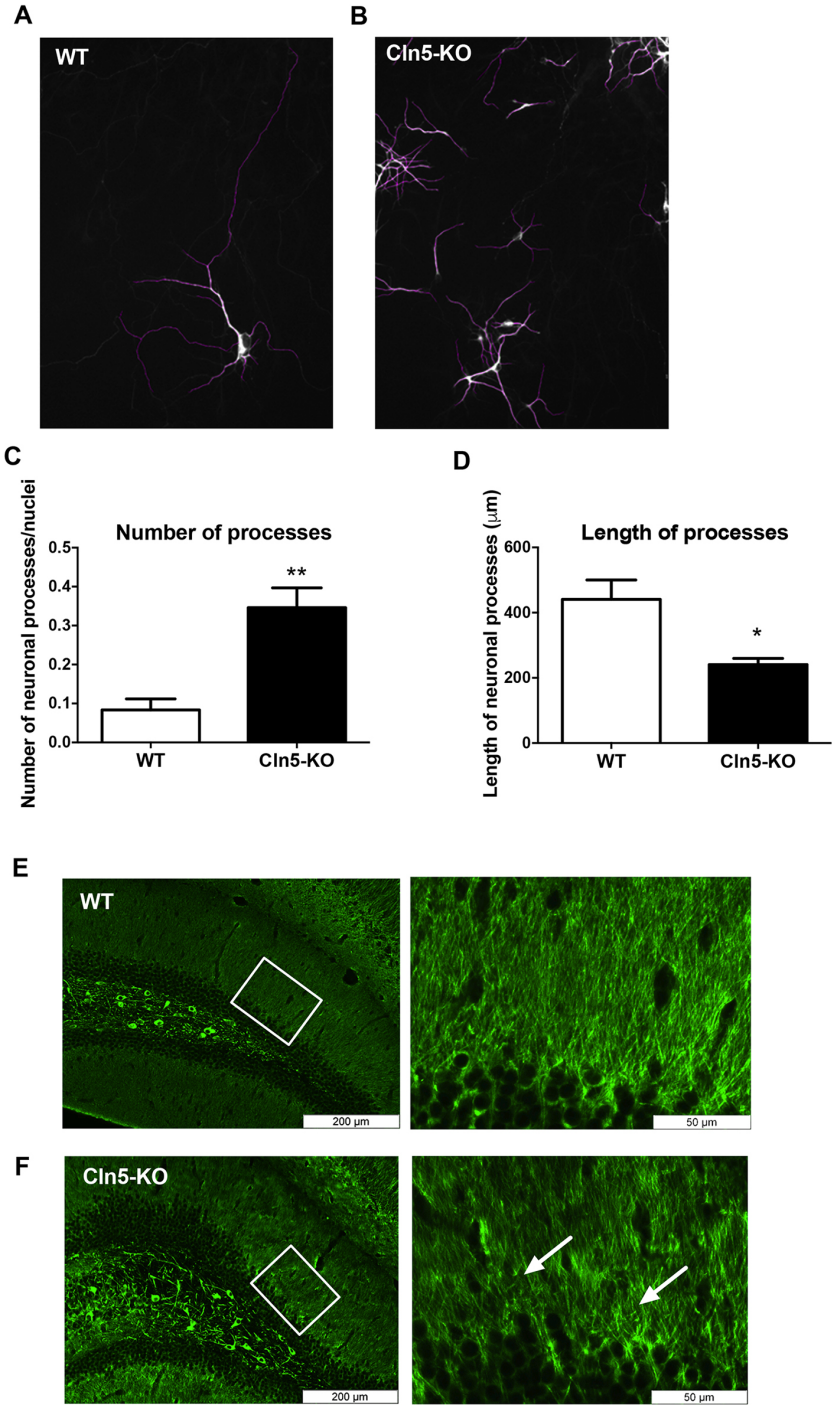
**Neuronal process length and number is altered in *Cln5*-deficient cells.** The numbers and lengths of neuronal processes in WT and *Cln5*-KO cells were visualized and quantified following immunostaining for Tuj-1. (A,B) Representative images of Tuj-1-positive WT and *Cln5*-KO cells. (C) The number of processes per nuclei were calculated from 4 replicate wells and are expressed as mean±s.d. **P<0.001. (D) The length of processes was calculated from 4 replicate wells and is expressed as mean length of neuronal processes in μm±s.d. *P<0.05. (E,F) Representative images of MAP-2 histochemistry in 7-month-old *Cln5*-KO and WT mouse hippocampi. The boxed area on the left is shown at higher magnification on the right. Arrows point to areas of interest where the morphology of neurites is altered. Statistical method: 2-tailed *t*-test.

Given that *Cln5* loss appeared to increase neuronal differentiation, but reduce cellular migration, we next explored the appearance of the cells in more detail by measuring the numbers and lengths of neuronal processes in Tuj-1-positive WT (Fig. 7A) and *Cln5*-KO (Fig. 7B) cells. In comparison to WT controls, the *Cln5*-deficent cells had an approximately 3-fold increase in the number of processes per nucleus (Fig. 7C). However, the mean length of each *Cln5*-KO neurite was reduced by over 40% (Fig. 7D). These data suggest that, even though the number of cells of the neuronal lineage and the lengths of neuronal processes are increased in *Cln5* deficiency, loss of *Cln5* results in impairment of the formation of mature neurons with long processes. In support of this data, an altered neuronal phenotype was observed in the hippocampi of *Cln5*-KO mice. Immunohistological staining for microtubule-associated protein 2 (MAP-2; a marker of neuronal cytokskeleton) in 7-month-old mouse brains revealed that the morphology of MAP-2-positive neuronal processes in the hippocampi was altered under *Cln5* deficiency (Fig. 7E,F).

Taken together, these results demonstrate that loss of *Cln5* induced the NPCs to differentiate towards the neuronal lineage, yet the migration and formation of neuronal processes was impaired.

### Absence of *Cln5* alters Ca^2+^ responses of differentiating neuronal progenitors

**Fig. 8.**
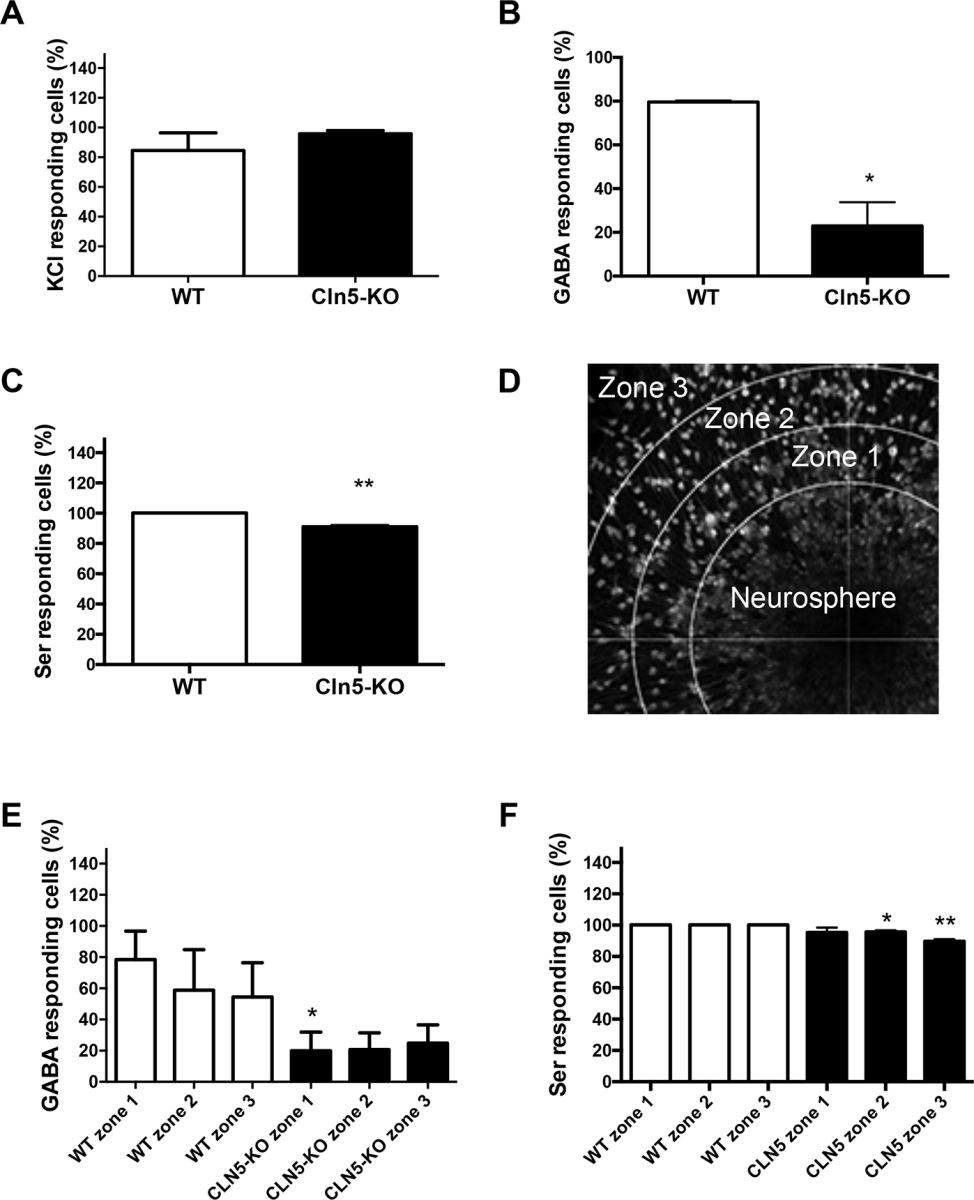
**Loss of *Cln5* alters Ca^2+^ responses of differentiating neuronal progenitors.** Confocal calcium imaging was applied to measure the responses of WT and *Cln5*-KO neuronal progenitors to neurotransmitters and high-potassium solution. (A) The percentage of cells responding to KCl-induced depolarization was calculated from the periphery of 3-4 neurospheres containing over 200 cells each and is expressed as Fluo-4 fluorescence intensity and the mean percentage of responding cells±s.e.m. (B) The percentage of cells responding to 100 μM GABA was calculated from the periphery of 3-4 neurospheres containing over 200 cells each and is expressed as the mean percentage of responding cells±s.e.m. *P*<0.05. (C) The percentage of cells responding to 100 μM serotonin (Ser) was calculated from the periphery of 3-4 neurospheres containing over 200 cells each and is expressed as the mean percentage of responding cells±s.e.m. **P<0.001. (D) Fluo-4 fluorescence image of the migration area that was divided into 3 zones. The cells in each migration area were divided into 3 groups based on the migration path length. (E) The percentage of cells responding to 100 μM GABA was calculated in each of the 3 migratory zones and is expressed as the mean percentage of responding cells±s.e.m. *P<0.05. (F) The percentage of cells responding to 100 μM serotonin was calculated in each of the 3 migratory zones and is expressed as the mean percentage of responding cells±s.e.m. *P<0.05, **P<0.001. Statistical method: 2-tailed *t*-test.

To test the hypothesis that the phenotypic changes observed in *Cln5*-deficient cells are concomitant with a functional impairment, we analyzed intracellular calcium (Ca^2+^) transients from the differentiated NPCs by using confocal Ca^2+^ imaging. Depolarization-induced intracellular calcium transients were analyzed from neurospheres that had been differentiated for 3-4 days. Neurospheres were treated with high-potassium solution to induce depolarization of neuronal cells. The majority of cells responded to depolarization-induced changes in calcium concentration and there were no genotype-related differences observed (Fig. 8A). The number of cells responding to GABA (Fig. 8B) and serotonin (Ser, Fig. 8C) were reduced upon *Cln5* deficiency. There was no genotype difference in the number of cells responding to ATP, acetylcholine or glutamate (data not shown). Previously, it has been shown that the calcium responses of NPCs may alter depending on the distance they migrate away from the edge of the neurosphere (Achuta et al., 2014). Therefore, for the analysis, we next divided the cell migration area into three zones starting from the edge of the neurosphere and reaching to the outermost migrated cells (Fig. 8D). When the data was analyzed separately for each migratory zone, the results for both GABA- and Ser-induced changes in intracellular calcium transients remained similar to that in Fig. 8B,C.

### Neurogenesis impairment in *Cln5* deficiency is associated with increased expression of IL-1β

**Fig. 9.**
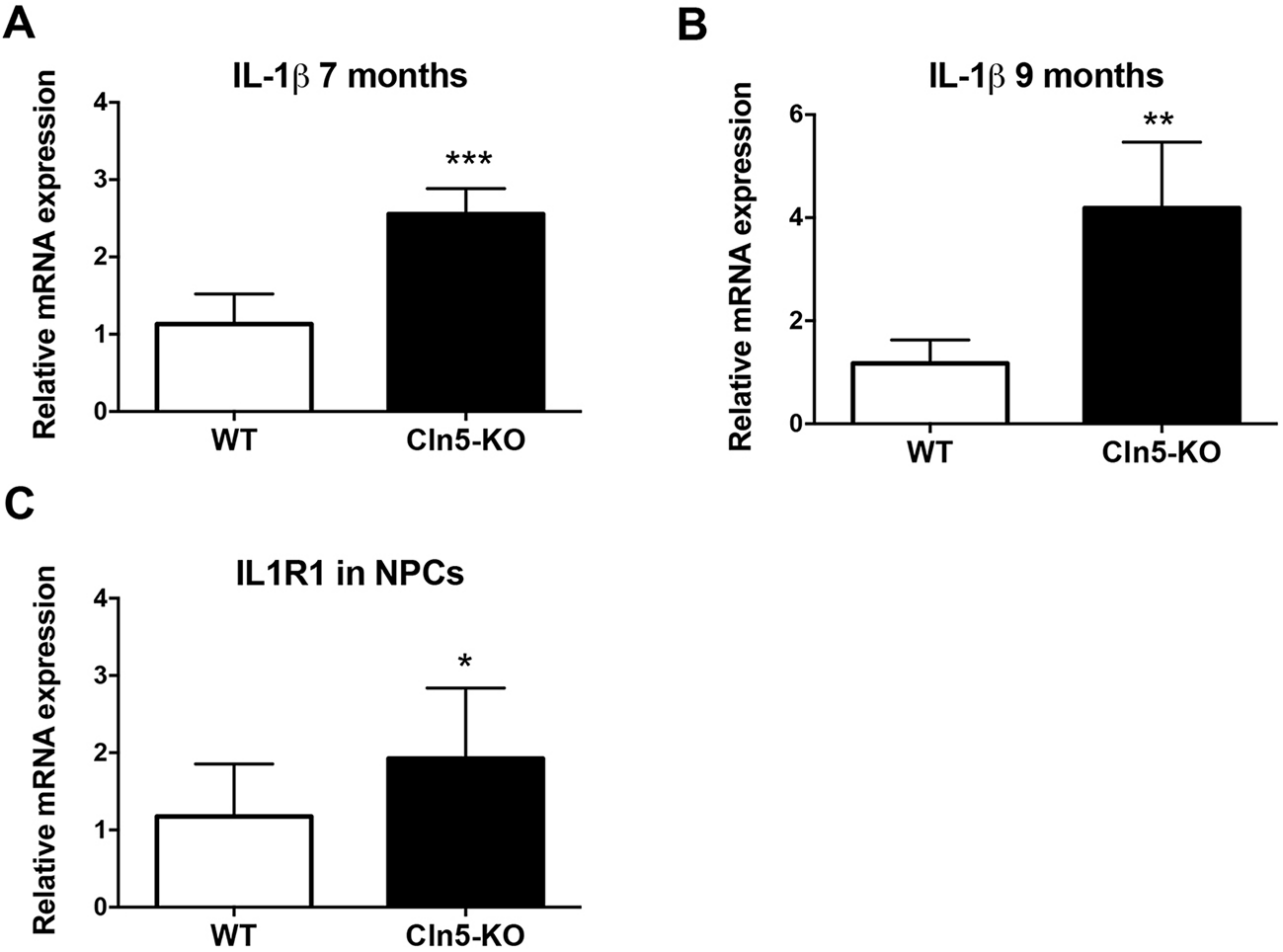
**IL-1β signaling is altered in *Cln5* deficiency.** (A,B) Relative expression of mRNA encoding IL-1β in the hippocampi of WT and *Cln5*-KO mice was analyzed by qRT-PCR (*n*=6-7/group). The mRNA expression level is presented as mean relative expression level normalized to GAPDH±s.d. **P<0.001, ***P<0.001. (C) Relative expression of mRNA encoding the IL-1β receptor (IL1R1) in WT and *Cln5*-KO neurospheres was analyzed by qRT-PCR (*n*=12-13/group). The mRNA expression level is presented as mean relative expression level normalized to GAPDH±s.d. *P<0.05. Statistical method: 2-tailed *t*-test.

Numerous molecules, growth factors and cytokines are involved in the regulation of neurogenesis. To assess whether the increased level of neurogenesis observed in *Cln5*-deficient brains occurs concomitantly with alterations in the expression of these factors, qRT-PCR analyses were carried out for components of the Wnt signaling pathway (catenin beta-1 and axin-1); the trophic factors nerve growth factor (NGF), brain-derived neurotrophic factor (BDNF), insulin growth factor 1 (IGF1) and tumor necrosis factor (TNF); and the cytokines interleukin (IL)-6 and IL-1β. The expression levels of the majority of factors measured remained unaltered in *Cln5* deficiency (data not shown). A modest, but statistically significant, 0.6-fold reduction was observed in IL-6 expression in the 9-month-old *Cln5*-KO hippocampi (data not shown). Likewise, a small, 0.3-fold, reduction in NGF expression was observed in the *Cln5*-deficient brains (data not shown). Interestingly, the expression of IL-1β was robustly increased in the hippocampi of both 7- and 9-month-old *Cln5*-KO mice (Fig. 9A,B). The increase of IL-1β in *Cln5*-deficient hippocampi was confirmed by ELISA (data not shown). The expression of IL-1β was not changed at the age of 3 months (data not shown), the age at which DCX expression was also unaltered in *Cln5*-KO mice. Loss of *Cln5* also did not alter the mRNA expression levels of the IL-1β receptor (IL1R1) or the receptor accessory protein in the hippocampi (data not shown). This finding of robust IL-1β elevation in *Cln5*-KO hippocampi that occurs concomitantly with neurogenesis alterations prompted us to further investigate the role of IL-1β in neurogenesis in CLN5 disease.

**Fig. 10.**
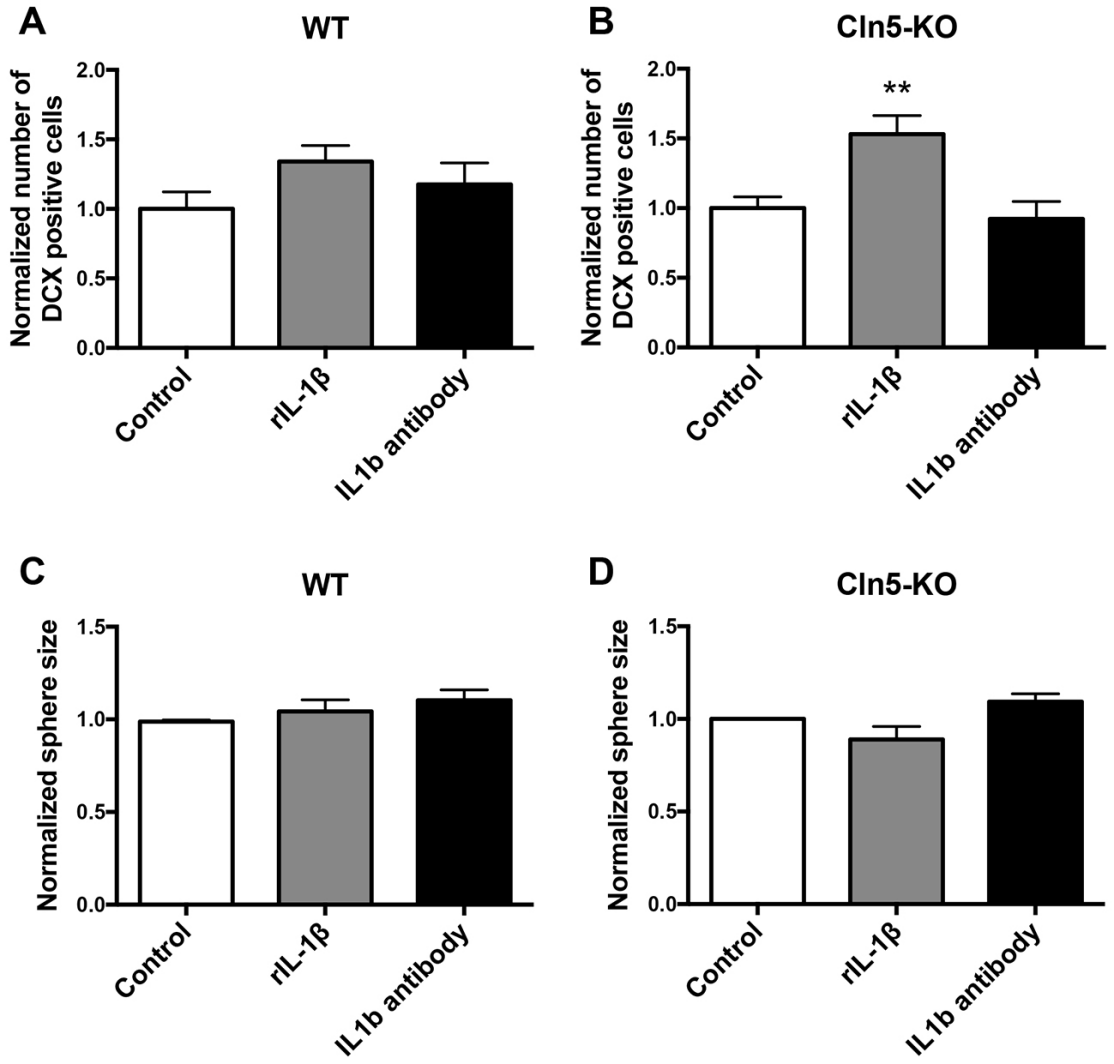
**Involvement of IL-1β in NPC differentiation and proliferation.** (A,B) NPCs were treated with 50 ng/ml recombinant IL-1β (rIL-1β) or with 1 μg/ml anti-IL-1β for 7 days and processed for immunocytochemistry. The DCX staining was quantified from 6-18 images each from the periphery of the neurosphere area where the NPCs grow separately. **P<0.001. (C,D) NPCs were cultured in the presence of 50 ng/ml rIL-1β or 1 μg/ml of anti-IL-1β for 7 days to form floating neurospheres. Proliferation was assessed by measurement of sphere size from 8-18 images, *n*=3-6. Data from multiple experiments are shown normalized to the size of spheres grown in control conditions. Statistical method: ordinary 1-way ANOVA.

To characterize the effects of IL-1β in NPCs *in vitro*, IL-1β levels were first measured by qRT-PCR and cytometric bead array (CBA) assay from WT and *Cln5*-KO neurospheres, and by enhanced-sensitivity CBA assay from the cell culture media. However, IL-1β levels were too low and below the detection limit of both methods. Nevertheless, loss of *Cln5* increased the expression level of IL1R1 in neurospheres (Fig. 9C), suggesting that this signaling pathway is altered in *Cln5*-KO NPCs. The expression of the IL-1 receptor accessory protein was not altered (data not shown). Next, to further investigate the relationship between IL-1β and neurogenesis in CLN5 disease, we treated WT and *Cln5*-KO NPCs with recombinant IL-1β, or with an IL-1β blocking antibody. Treating WT NPCs with recombinant IL-1β for 7 days did not affect the number of DCX-positive cells (Fig. 10A). However, a significant increase in the number of DCX-positive cells was observed in *Cln5*-KO cultures (Fig. 10B). Treatment with the IL-1β blocking antibody had no effect in either WT or *Cln5*-KO cultures. Treatment of NPCs with recombinant IL-1β or the anti-IL1β antibody did not affect sphere size in either WT (Fig. 10C) or *Cln5*-KO (Fig. 10D) cultures.

## DISCUSSION

Our findings demonstrate a newly identified role for *Cln5* in neurogenesis. We report for the first time that neurogenesis is increased in *Cln5*-KO mice, which model the childhood neurodegenerative disorder caused by *CLN5* deficiency. These results demonstrate that, during *Cln5* deficiency, proliferation of NPCs is increased, NPC migration is reduced and NPC differentiation towards the neuronal lineage is increased concomitantly with functional alterations in the NPCs. Moreover, the observed impairment in neurogenesis is correlated with increased expression of the pro-inflammatory cytokine IL-1β.

To our knowledge, this paper is the first description of impaired neurogenesis of the mammalian brain in NCL. The increase in neurogenesis did not occur at the age of 3 months, but was observed starting at the age of 5 months. At this age, it is known that *Cln5*-KO mice show such pathological features as glial activation and defective myelination (Schmiedt et al., 2012). A previous study has shown that secondary neurogenesis is impaired in a zebrafish model of CLN2 disease (Mahmood et al., 2013), yet information about NCL-related alterations in mammalian neurogenesis is completely lacking. Recently, Fabritius et al. described that, during embryonic brain development, *Cln5* is strongly expressed in the hippocampi and ventricular region (Fabritius et al., 2014). This high expression in those brain regions where neurogenesis is known to occur continued into the early postnatal period and suggested that *Cln5* may have a role in the generation of new neurons during embryonic development. Our results are in agreement with this suggestion and demonstrate that loss of *Cln5* impairs neurogenesis *in vivo* and has striking effects on the phenotype of NPCs *in vitro*.

In support of our *in vivo* and *in vitro* results of increased numbers of neuronal precursor cells in *Cln5* deficiency, alterations in cell proliferation have also been demonstrated to occur in the brains of other NCL mouse models. For example, Weimer et al. showed that, in the early postnatal cerebella, the proportion of proliferating cells is increased in *Cln3* deficiency (Weimer et al., 2009). In addition, neuronal progenitors derived from the ventricular wall of *Cln1*-deficient mice were also shown to proliferate more than WT cells, whereas alterations in their ability to differentiate were not observed (Ahtiainen et al., 2007). Given that impaired proliferation of precursor cells is observed in at least three forms of NCL, it is possible that these alterations are a common disease feature of the NCLs. Further studies are clearly required to assess how various aspects of neurogenesis are affected in multiple forms of the disease.

The maturation of neurons has previously been shown to be reduced in NCL caused by loss of the CLN6 protein (Benedict et al., 2009). Using primary hippocampal neurons from the *nclf* mouse model of CLN6 disease, Benedict et al. demonstrated that cellular and synaptic connections were reduced in *Cln6*-deficient neurons. The neurons also displayed an abnormal morphology with thinner processes when compared to WT cells. Interestingly, neuronal growth cone and axon guidance pathways are affected in the *Cln5*-KO mouse model (von Schantz et al., 2008). Our results support these findings of altered neuronal morphology. The neuronal processes of differentiated *Cln5*-KO cells were shorter than those of WT cells, yet the number of processes was increased in the *Cln5*-deficient cells. In addition, the migration of *Cln5*-deficient cells was reduced and their differentiation towards the neuronal lineage was increased. It may well be that our observed defects are related to impaired neuronal maturation and at least partly explain the increase in neurogenesis under *Cln5* deficiency. It is possible that increased NPC proliferation and differentiation towards the neuronal lineage is a compensatory mechanism to counteract the loss of fully functional, mature neurons in the *Cln5*-KO brain. Our results also suggested that alterations in neurogenesis that are caused by *Cln5* deficiency are not related to changes in hippocampal apoptosis. However, in addition to apoptosis, other mechanisms, such as autophagy, have been reported to modulate stem cell development and neurogenesis (Wu et al., 2016; Li et al., 2016). Given that autophagy impairment is a central feature of the NCLs, it is possible that alterations in neurogenesis and autophagy are linked and should be further investigated.

The idea that proliferation and differentiation of neural stem cells are a convergence point for several neurodevelopmental disorders has gained support in recent years (Ernst, 2016). It has been hypothesized that many genes associated with these disorders may function as brakes on stem cell differentiation, which leads to inappropriate interpretation of environmental signals. In the current study, we utilize a widely used cellular model of neurogenesis, NPCs, harvested from the hippocampi of embryos at embryonic day 18 (E18). We report that proliferation, migration and differentiation are all altered at this early age in the *Cln5*-deficient mice. Furthermore, we describe an increase in the number of newly born neurons in the hippocampus in adult animals. These results suggest that the onset of neurogenesis deficits occur early in the development in *Cln5* deficiency and are also observed in adulthood.

Our calcium imaging data supports the notion that the neuronal progenitor cells are functionally altered in *Cln5* deficiency. This data revealed functional changes in the response of *Cln5*-deficient cells to serotonin and GABA. Developmental impairments of select neurotransmitter systems have been described in the brains of *Cln3*-deficient mice (Herrmann et al., 2008), yet no information has previously been available for CLN5 disease. Deregulation of the serotonin system is known to be associated with neurogenic alterations and serotonin has been shown to have a neurogenic role in the hippocampus (Alenina and Klempin, 2015). In our study, the percentages of *Cln5-*KO NPCs responding to serotonin were slightly reduced when compared to WT cells. Interestingly, populations of GABAergic interneurons are affected in several NCL forms, both in humans and disease models (Oswald et al., 2008; Tyynelä et al., 2004; Pears et al., 2007). To our knowledge, this is the first report of reduced GABA response in NCL cultures that are harvested from embryonic brains, thereby suggesting that alterations to GABA responses are already altered at a very early stage in disease progression in *Cln5-*KO NPCs.

The exact function of the CLN5 protein has remained unknown, although it has been reported to be associated with lipid metabolism, myelination, protein transport and endosomal sorting (Cárcel-Trullols et al., 2015). It is known that the *Cln5* gene is widely expressed in brain and peripheral tissues (Heinonen et al., 2000; Holmberg et al., 2004), and *Cln5* expression has been shown to be higher in microglia than in neurons (Schmiedt et al., 2012). *Cln5* is also expressed in cortical embryonic neurospheres (Fabritius et al., 2014). We demonstrate that *Cln5* is expressed in hippocampus-derived NPCs and show that its relative expression level in neurospheres is similar to that in microglia. This relatively high expression level suggests an important role for *Cln5* in NPCs and supports the idea that *Cln5* is important for neurogenesis.

Neuroinflammatory processes associated with neurodegenerative diseases are known to affect neurogenesis. In particular, microglia have a fundamental role in regulating the generation of neuronal cells within neurogenic niches through phagocytosis and cytokine production (Shigemoto-Mogami et al., 2014; Cunningham et al., 2013). Therefore, factors that alter the number or activation state of microglia can have a profound effect on neurogenesis. Neuroinflammation and microglia activation is a well-known pathological feature of many neurodegenerative diseases, including CLN5 disease (Schmiedt et al., 2012; Tyynelä et al., 2004). The activated microglia produce several pro-inflammatory cytokines, including IL-1β. Although it is generally believed that pro-inflammatory cytokines inhibit neurogenesis, whereas anti-inflammatory cytokines support it, the role of cytokines, and IL-1β in particular, in neurogenesis remains highly debatable (Molina-Holgado and Molina-Holgado, 2010). For example, IL-1β has been shown to both increase (Peng et al., 2008; Seguin et al., 2009) and reduce (Guadagno et al., 2015) the proliferation of NPCs. Hippocampal neurogenesis has been shown to be impaired in mice that are chronically exposed to IL-1β (Koo and Duman, 2008), and suppression of microglia activation has been shown to reduce the production of IL-1β and inhibit neurogenesis (Shigemoto-Mogami et al., 2014).

One mechanism through which neurogenesis appears to be regulated in CLN5 disease involves IL-1β. The expression of IL-1β was high in the hippocampi of *Cln5*-KO mice, the area in which neurogenesis was increased. In NPCs, the expression levels of this cytokine were too low for detection. The addition of exogenous IL-1β to the cultures resulted in an increase in the proportion of *Cln5*-KO cells directed towards the neuronal lineage. IL-1β exerts its effects on target cells by binding to IL1R1, which is expressed on NPCs (Wang et al., 2007). It is known that IL-1β treatment causes upregulation of IL1R1 expression on NPCs (Green et al., 2012). The fact that the expression of IL1R1 was elevated in *Cln5*-KO NPCs supports the notion of altered IL-1β signaling in CLN5 disease. However, in addition to IL-1β, IL1R1 is bound by IL-1α, which is also known to affect NPC fate (Ajmone-Cat et al., 2010). Our studies show that specific blocking of IL-1β alone in NPC cultures did not affect their differentiation towards the neuronal lineage. It is possible that blocking only IL-1β with the antibody treatment is not sufficient to block signaling through the receptor complex and a more robust approach of blocking this complex may have produced different results. Nevertheless, our data demonstrate alterations in IL-1β signaling during *Cln5* deficiency. Altered IL-1β signaling has also been demonstrated to occur in common neurodegenerative diseases such as Alzheimer's disease (Alam et al., 2016), in which neurogenic changes have also been reported (Winner and Winkler, 2015).

Approximately 5% of the CLN gene mutations have been associated with adult-onset neurological phenotypes (Mancini et al., 2015). Although most *CLN5* mutations result in disease onset between the ages of 4 and 7 years, some mutations are associated with juvenile to early-adult onset (Cannelli et al., 2007; Xin et al., 2010). The identified mutations include missense, splicing and nonsense alterations. More recently, a newly identified mutation in *CLN5* was reported to result in the onset of neurological manifestations after the age of 50 (Mancini et al., 2015). Based on the heterogeneous age of disease onset, it is possible that the different mutations cause distinct alterations to the function of the CLN5 protein. The neurogenesis alterations observed in this study occur upon disruption of exon 3 of the mouse *Cln5* gene, which results in a frameshift and premature stop codon in exon 4 (Kopra et al., 2004). Therefore, further studies should focus on deciphering mechanisms of neurogenesis in cells from patients with CLN5 disease.

We demonstrate for the first time that neurogenesis in the mammalian brain is altered in NCL and show the importance of the CLN5 protein for this process. Increased neurogenesis is associated with high levels of IL-1β in the diseased brains, and alterations to IL-1β signaling in NPCs *in vitro*. A full understanding of the pathological mechanisms that lead to disease and the function of the NCL proteins are critical for designing effective therapeutic approaches for this devastating neurodegenerative disorder.

## MATERIALS AND METHODS

### Animal models

All experimental procedures were carried out according to the national regulation of the usage and welfare of laboratory animals and approved by the Animal Experiment Committee in State Provincial Office of Southern Finland. *Cln5-*KO mice of both genders with a deletion of exon 3 in the *Cln5* gene (Kopra et al., 2004), kindly provided by Dr Anu Jalanko, were used for the study. Age-matched WT C57BL/6JRccHsd mice were used as controls.

### BrdU administration

BrdU (Sigma-Aldrich, Louis, MO, USA) was administered to 5-month-old *Cln5*-KO and WT mice intraperitoneally (i.p.) at a dose of 50 mg/kg body weight every 12 h during 7 consecutive days. The BrdU stock solution was prepared in phosphate buffered saline (PBS), and the pH was adjusted to 7.1-7.4 with 10 M NaOH. Mice were sacrificed and the brains further processed for histology as described below.

### Immunohistochemistry

The mice were deeply anesthetized with avertin and perfused with heparinized saline. The brains were collected, post-fixed with 4% paraformaldehyde (PFA) overnight and cryoprotected with 30% sucrose (VWR International, Leuven, Belgium) solution for 48 h. The brains were snap-frozen in liquid nitrogen and cut to 20-μm sections with a cryostat (Leica Microsystems GmbH, Wetzlar, Germany). Following blocking with 10% normal goat serum (NGS; Chemicon International, Merck Millipore, Billerica, MA, USA), 5-7 sections 400-μm apart were incubated overnight with DCX (Cell Signaling, Danvers, MA, USA), MAP-2 (Merck Millipore, Billerica, MA, USA) and Ki-67 (Abcam, Cambridge, UK) primary antibodies at 1:200 or 1:500 dilution. Following washing, sections were incubated for 2 h with an Alexa-Fluor-488 or Alexa-Fluor-568 secondary antibody (Molecular Probes, Invitrogen, Eugene, OR, USA). For BrdU staining, sections were permeabilized in an ethanol–acetic-acid mixture, after which DNA denaturation was carried out by treatment in 2N HCl for 30 min at 37°C. HCl was neutralized by treatment in 0.1 M borate buffer pH 8.5 for 10 min at room temperature (RT). After washing with PBST, the sections were treated with mouse IgG blocking reagent (Vector Laboratories, CA, USA) followed by blocking in 0.1% bovine serum albumin/5% NGS. PBST-rinsed sections were then incubated with BrdU primary antibody (Roche, Basel, Switzerland) at 1:50 dilution overnight at RT. Following washing, sections were incubated for 2 h with an Alexa-Fluor-568 secondary antibody (Molecular Probes, Invitrogen). Stained sections were visualized using an Olympus AX70 microscope (Olympus Corporation, Tokyo, Japan) equipped with a digital camera (Color View 12 or F-Fiew; SoftImaging Systems, Gmbh, Munster, Germany). The numbers of immunoreactive cells were counted and/or the immunoreactive areas were quantified using ImagePro Plus software (Media Cybernetics, Rockville, MD, USA).

### Quantitative real-time polymerase chain reaction

Total RNA was isolated using RiboPure Kit or Trizol reagent (both from Thermo Fisher Scientific, Waltham, MA, USA) according to the manufacturer's instructions. The purity and concentration of RNA was measured with a Nanodrop 1000 spectrophotometer (Thermo Fisher Scientific). Five-hundred nanograms of total RNA were used for cDNA synthesis using random hexamer primers (Promega, Madison, WI, USA) and Maxima reverse transcriptase (Fermentas, Thermo Fisher Scientific). The relative expression levels of mRNA were measured according to the manufacturer's protocol by qRT-PCR (StepOnePlus; Life Technologies Carlsbad, CA, USA) using TaqMan chemistry and specific assays-on-demand target mixes (Life Technologies, Carlsbad, CA, USA). The expression levels were obtained by normalizing the target gene to ribosomal RNA or to *GAPDH*, and presented as fold change in the expression or in arbitrary units using the 2^-ΔΔCt^ method; Ct is the threshold-cycle value.

### NPC culture

The hippocampi were dissected from the brains of WT and *Cln5*-KO mice at E18 and cultured as described (Kärkkäinen et al., 2014) as free-floating neurospheres in the presence of epidermal growth factor (EGF) and basic fibroblastic growth factor (FGF). Cells were grown in culture medium containing DMEM/F12 (Gibco, Thermo Fisher Scientific), 1 M HEPES (Sigma-Aldrich), 100 U/ml penicillin, 100 mg/ml streptomycin, B27 supplement (all from Gibco, Thermo Fisher Scientific), 20 ng/ml EGF (PeproTech, Rocky Hill, NJ, USA) and 10 ng/ml basic FGF (PeproTech). Cells were cultivated at 37°C in 5% CO_2_. Fresh medium as well as EGF and FGF were added to the cells every 3 days.

### Characterization of NPC proliferation, migration and differentiation

To quantify NPC proliferation, cells were plated at a density of 30,000 cells in 6-well plates in culture media supplemented with EGF and FGF. After 3-7 days, neurospheres were imaged and the average size of neurospheres was measured using ImageJ (Wayne Rasband, National Institutes of Health, Bethesda, MD, USA) software. In addition, proliferation was assessed by BrdU labeling. NPCs were incubated in culture media supplemented with EGF, FGF and 10 µM BrdU (Sigma-Aldrich) for 24 h. After incubation, cell clusters were dissociated using TryplE (Gibco, Thermo Fisher Scientific) and single NPCs were applied onto poly-DL-ornithine (Sigma-Aldrich)-coated 48-well plates overnight in culture media without growth factors. On the next day the cells were fixed with 4% formaldehyde solution and immunostained as described below. Cells were imaged with an Olympus IX71 microscope with MT10 illumination system attached with the DP70 digital camera, running DP software (all from Olympus, Tokyo, Japan) and BrdU-positive cells were counted from replicate wells.

To assess NPC migration, 10-15 neurospheres were collected and plated onto the bottom of poly-DL-ornithine (Sigma-Aldrich)-coated plates and grown for 7 days without growth factors. For the evaluation of cell migration, neurospheres were imaged as above and the average migration distance from the edge of the neurosphere was measured using ImagePro Plus (Media Cybernetics, Rockville, MD, USA) or ImageJ (Wayne Rasband, National Institutes of Health, Betheda, MD, USA) software.

To measure the differentiation of NPCs, neurospheres were plated onto the bottom of poly-DL-ornithine-coated plates as above and grown for 3 or 7 days without growth factors. Neurospheres were then fixed with 4% formaldehyde solution and immunostained for GFAP (Dako, Glostrup, Denmark), Tuj-1 (Covance, Princeton, NJ, USA) or DCX (Cell Signaling) as described below. Samples were imaged and quantified using ImagePro Plus software (Media Cybernetics, Rockville, MD, USA). Fiji (an image-processing package based on ImageJ) and neurite tracer plugin were used for the neurite analyses.

### Treatment of NPCs

To investigate the relationship between IL-1β and NPC proliferation and differentiation, WT and *Cln5*-KO neurospheres were dissociated to single cells and then grown for 6 days in the presence of anti-IL1β or control IgG antibody (both 1.0 μg/ml, Abcam, Cambridge, UK). In another set of experiments, NPCs were grown in the presence of 50 ng/ml recombinant IL-1β (PeproTech) for 6-9 days. Assessment of the effect of IL-1β blockage and treatment with recombinant IL-1β on NPC proliferation and differentiation were assessed as above.

### Primary microglia culture

Primary mouse microglia were isolated and prepared from mouse pups at the age of P0-P1 as described previously (Hanisch et al., 2004). After brain isolation, meninges were removed and brain tissues were digested with 0.05% trypsin (Sigma-Aldrich) and ethylenedinitrilotetraacetic acid (EDTA; Merck, Kenilworth, NJ, USA) and incubated in a humidified atmosphere at 37°C in 5% CO_2_ for 20 min. Cells were suspended in DMEM (Gibco, Thermo Fisher Scientific) supplemented by 10% FBS, 100 U/ml penicillin-streptomycin, 2 mM L-glutamine (all from Gibco, Thermo Fisher Scientific) and plated onto cell culture flasks for cultivation. At 12 days after cultivation, microglia cells were harvested by shaking or by trypsinization. Cells were plated on 12-well plates and used for RNA isolation 2 days after plating.

### Immunocytochemistry

To characterize cell phenotype, fixed cells were permeabilized with ice-cold methanol (Thermo Fisher Scientific) for 20 min and blocked for non-specific binding with 10% NGS (Chemicon International, Merck Millipore) in PBS for 2 h. After that, cells were incubated with primary antibody diluted in 5% NGS in PBS for 24 h at +4°C. Then, the cells were washed 3 times with PBS and incubated with Alexa-Fluor-488 or -568 secondary antibodies (Molecular Probes, Invitrogen) diluted in 5% NGS in PBS for 2 h at RT and protected from light. Following washing, cell nuclei were stained with 2.5 μg/ml bisBenzimide (Hoechst; Sigma-Aldrich) for 5 min. For BrdU staining, before blocking of non-specific sites the cells were incubated with 2 N HCl for 10 min, washed again and then stained using the protocol described above.

### Confocal calcium imaging

Calcium imaging was performed as previously described (Mutikainen et al., 2016). Following 3-4 days of differentiation and growth on poly-DL-ornithine-coated coverslips, NPCs were incubated with 5 μM of Fluo-4-acetoxymethyl (AM)-ester (Thermo Fisher Scientific), an intracellular calcium indicator, at 37°C for 30 min. Then, coverslips with attached neurospheres were transferred to the recording chamber (Cell MicroControls) with flow rate approximately 1-2 ml/min. For perfusion, preheated and oxygenated (37°C, 100% O_2_) external saline solution containing 125 mM NaCl, 5.5 mM KCl, 2 mM CaCl_2_, 0.8 mM MgCl_2_, 10 mM HEPES, 24 mM glucose and 36.5 mM sucrose (pH 7.3) was used. Measurement was carried out after a period of 20 min to allow de-esterification of the dye. Measurement was performed with a confocal inverted microscope FluoView 1000 (Olympus, Japan). To measure calcium signals, the cells were excited at 488 nm, the emitted light band was 500-600 nm through a 20× objective lens, and frame-scan mode was used at resolution 800×800. Fluo-4 fluorescence intensity is expressed as an *F*/*F*_0_ - ratio, where *F* is the background subtracted fluorescence intensity and *F*_0_ is background subtracted minimum fluorescence value measured from each cell at rest. For chemical stimulation, 100 μM acetylcholine, 100 μM glutamate, 100 μM serotonin, 100 μM ATP (all from Sigma-Aldrich) and high-potassium solution containing (in mM) 5.5 NaCl, 100 KCl, 2 CaCl_2_, 0.8 MgCl_2_, 10 HEPES, 24 glucose and 36.5 sucrose (pH set at 7.3) were applied directly to the studied area with local perfusion manifold (Cell MicroControls).

### Statistical analyses

The data were analyzed using *t*-test or 1-way ANOVA with Tukey's *post hoc* test as appropriate in GraphPad Prism software (GraphPad Software Inc., La Jolla, USA). A *P*-value of <0.05 was considered significant. Data are shown as means±s.d. and means±s.e.m. for NPC *in vitro* data.
